# Characterization of metal binding of bifunctional kinase/phosphatase AceK and implication in activity modulation

**DOI:** 10.1038/s41598-019-45704-z

**Published:** 2019-06-24

**Authors:** Xiaoying Zhang, Qingya Shen, Zhen Lei, Qianyi Wang, Jimin Zheng, Zongchao Jia

**Affiliations:** 1 College of Chemistry, Beijing Normal University, Beijing, 100875 China; 2 Department of Biomedical and Molecular Sciences, Queen’s University, Kingston, Ontario K7L3N6 Canada

**Keywords:** X-ray crystallography, Kinases

## Abstract

A unique bifunctional enzyme, isocitrate dehydrogenase kinase/phosphatase (AceK) regulates isocitrate dehydrogenase (IDH) by phosphorylation and dephosphorylation in response to nutrient availability. Herein we report the crystal structure of AceK in complex with ADP and Mn^2+^ ions. Although the overall structure is similar to the previously reported structures which contain only one Mg^2+^ ion, surprisingly, two Mn^2+^ ions are found in the catalytic center of the AceK-Mn^2+^ structure. Our enzymatic assays demonstrate that AceK-Mn^2+^ showed higher phosphatase activity than AceK-Mg^2+^, whereas the kinase activity was relatively unaffected. We created mutants of AceK for all metal-coordinating residues. The phosphatase activities of these mutants were significantly impaired, suggesting the pivotal role of the binuclear (M1-M2) core in AceK phosphatase catalysis. Moreover, we have studied the interactions of Mn^2+^ and Mg^2+^ with wild-type and mutant AceK and found that the number of metal ions bound to AceK is in full agreement with the crystal structures. Combined with the enzymatic results, we demonstrate that AceK exhibits phosphatase activity in the presence of two, but not one, Mn^2+^ ions, similar to PPM phosphatases. Taken together, we suggest that metal ions help AceK to balance and fine tune its kinase and phosphatase activities.

## Introduction

The reversible phosphorylation and dephosphorylation modification of proteins with kinases and phosphatases primarily act on serine, threonine, and tyrosine residues, and is found in both prokaryotes and eukaryotes, where it regulates key signaling pathways in cells^1^. Opposite to kinases, protein phosphatases remove phosphate groups from proteins. Serine/threonine phosphatases can be categorized into three major families: PPPs (phosphoprotein phosphatases), PPMs (metal-dependent phosphatases), and the FCP/SCP family (aspartate-based phosphatases)^2–4^. Members of the PPP family normally consist of a catalytic subunit and a regulatory subunit or domain. Human PP1, PP2A, PP2B, and PP5 are the most representative eukaryotic members of this family^5,6^. The PPM family are metal-dependent enzymes containing manganese/magnesium ions (Mn^2+^/Mg^2+^), which are coordinated by a universally conserved core of aspartate residues. The PP2C phosphatases (the type 2 C of protein phosphatases) are typical representations of the PPM family^7–9^. Similar to members of the PPP family, metal ions can activate a water molecule for the dephosphorylation reaction in PP2C phosphatases^10,11^. In contrast to PPP and PPM families, FCP/SCP family rely on aspartic acids in the dephosphorylation process^12^.

In both Gram-positive and Gram-negative bacteria, phosphorylation of serine, threonine, and tyrosine residues has been observed^13–15^. Previous work has found that bacteria contain a number of PPM-like Ser/Thr phosphatases, revealing that Ser/Thr/Tyr phosphorylation and dephosphorylation play an important role in the prokaryotic life cycle^14^. In *Escherichia coli*, isocitrate dehydrogenase kinase/phosphatase (AceK) is a unique bifunctional enzyme with both protein kinase and phosphatase activities. AceK phosphorylates and dephosphorylates isocitrate dehydrogenase (IDH) in response to nutrient availability^16,17^. The reversible phosphorylation results in the inactivation or activation of IDH, which regulates the branch point between the Krebs cycle and the glyoxylate bypass^18–20^. Moreover, AceK exhibits an unusually strong ATPase activity compared to its kinase and phosphatase activities^21,22^. As the first example of prokaryotic phosphorylation identified, the crystal structures of AceK both alone and in complex with the IDH substrate have been determined^23–25^. The structure of AceK is similar to the canonical eukaryotic protein kinase, containing both kinase and regulatory domains^26,27^. However, how AceK with a kinase scaffold possesses phosphatase activity is not clear. As a protein phosphatase, AceK and PPMs share more conserved aspartic acids in the catalytic center despite the fact that the phosphatase function of AceK is strictly ATP/ADP-dependent. These conserved aspartic acids form hydrogen bonds with metal ion^23,27^. Previous studies have only shown one Mg^2+^ ion in the crystal structures of AceK, which is atypical because protein phosphatases usually contain two or more metal ions^4,23^. In light of the fact that only one metal was observed in AceK, it was hypothesized that AceK represents a novel type of phosphatase that distinguishes it from PPMs or PPPs; and the critical roles of the single Mg^2+^, ADP and Asp477 in the dephosphorylation were suggested^28^. Interestingly, recent theoretical studies showed that the presence of a single Mg^2+^ ion would lead to a higher energy barrier pathway in the phosphatase reaction than the double Mg^2+^ ions model, whereas the single metal model would lead to a lower energy barrier pathway in the kinase reaction^28,29^. This observation suggests that the metal ion in the catalytic center may have profound but opposing effects on kinase and phosphatase activities.

In this work, we report the crystal structure of AceK in complex with Mn^2+^ and ADP. Two Mn^2+^ ions were found in the active site of AceK, which is different from the previous structures. Results of the enzymatic activity of AceK with Mg^2+^ and Mn^2+^ revealed that the phosphatase activity of AceK-Mn^2+^ was higher than that of AceK-Mg^2+^, whereas the kinase activity was almost unchanged. We further characterized the catalytic role of the two metal ions by specifically mutating their surrounding residues. The phosphatase activity of both D477A and D477K were decreased, which coordinate the second metal ion (M2), demonstrating that metal ion binding at the M2 site contributes to phospho-substrate binding. Our ITC data indicate that Mn^2+^ and Mg^2+^ have different binding ratios (two and one respectively) for AceK WT in the presence of ADP and AMP. In addition, the binding affinity of Mn^2+^ for AceK WT was higher than that of Mg^2+^. The results from D477 mutants show that AceK displays activity in the presence of two Mn^2+^ ions, but not one. Taken together, our study reveals the catalytic role of binuclear metal centers, which helps to understand the reversible reaction catalyzed by the shared active site as well as the regulation of a delicate balance between the kinase and phosphatase activities of AceK.

## Results

### Overall structure and bound metal

**Figure 1:**
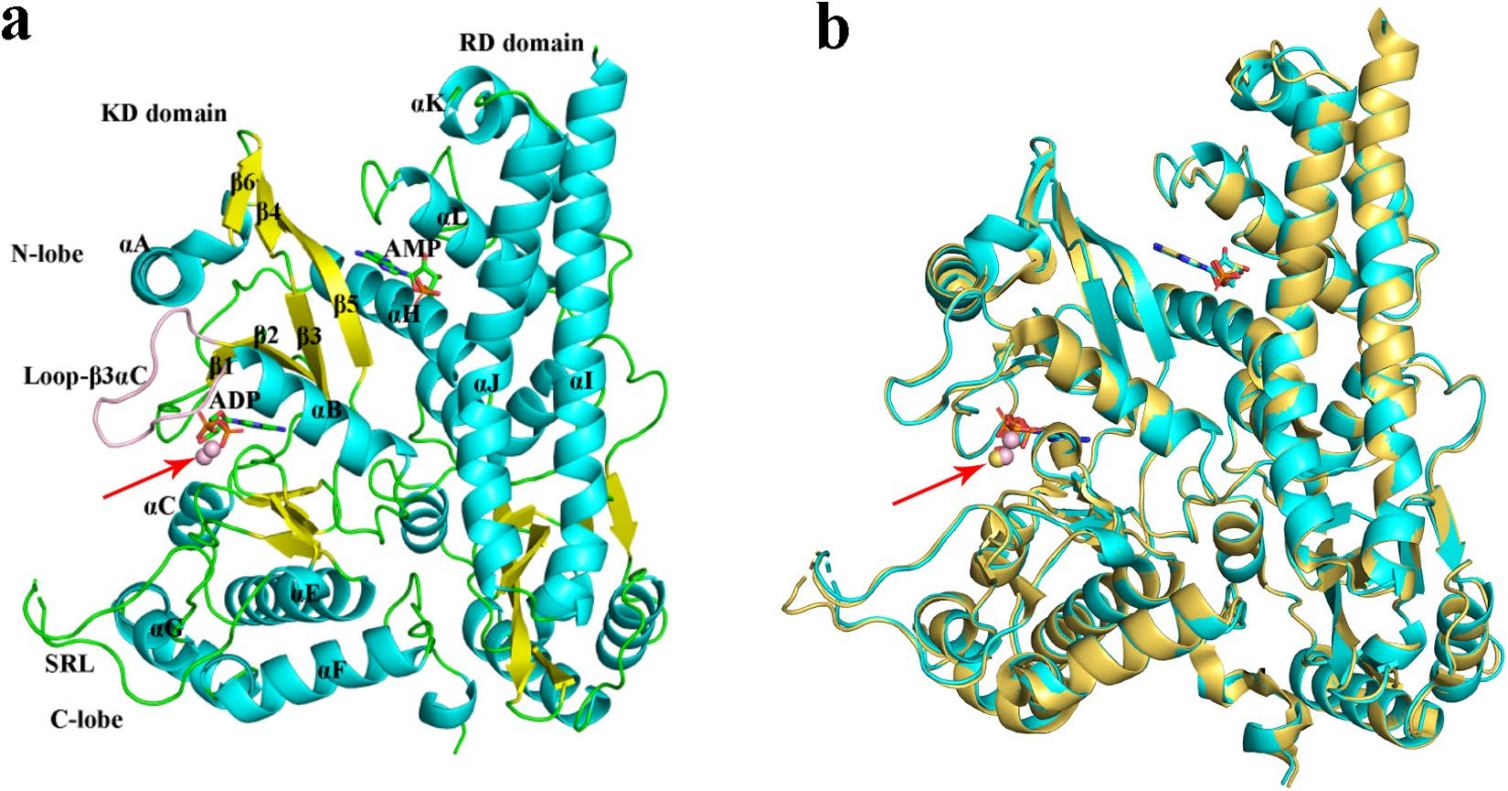
(**a**) The overall structure of AceK-Mn^2+^. The active site of AceK includes a buried ADP molecule and a bound AMP at the interface of the two domains. The kinase domain (KD) on the left resembles eukaryotic protein kinases. The regulatory domain (RD) on the right does not have any structural homologues. Loop-β3αC is coloured pink. (**b**) Structural comparison of AceK with Mn^2+^ (pink) and Mg^2+^ (light yellow). The location of metal ions is indicated by a red arrow.

We solved the crystal structure of AceK in complex with Mn^2+^ and ADP at 2.55-Å resolution (Table S1) by molecular replacement using the model of AceK in the complex of AceK-IDH (PDB:3LCB). The structure of AceK is composed of two distinct domains (Fig. 1a). The C-terminal part (kinase domain, KD) forms a typical kinase scaffold responsible for the functions of kinase, phosphatase and ATPase, which binds to adenosine triphosphate (ATP) or adenosine diphosphate (ADP). The KD of AceK is further divided into two lobes: the N-terminal lobe (N-lobe) predominantly forms β-sheet and the larger C-terminal lobe (C-lobe) mainly contains α-helices. It is shown that ATP/ADP is bound at the interface between the N-terminal and C-terminal lobes and is shielded by an extended loop (loop-β3αC)^23^. The N-terminal part known as regulatory domain (RD) of AceK is mainly composed of α-helices (Fig. 1a). In between KD and RD, there exists a pocket where AMP is bound to exert allosteric regulation of the catalytic activity in KD.

**Figure 2:**
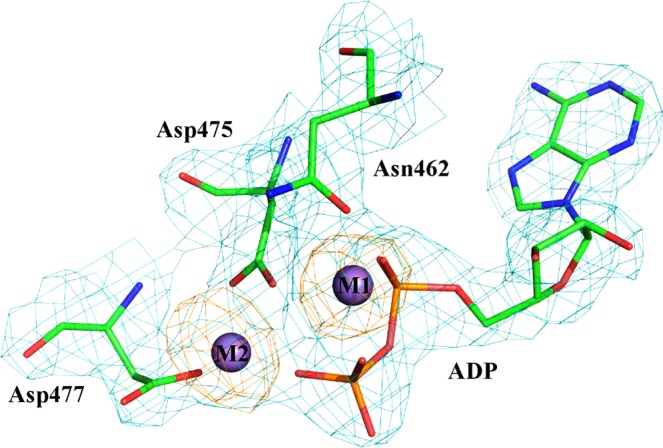
Electron density (2*mF*_*o*_ − *DF*_*c*_) map (contoured at 2σ, in cyan) at the catalytic center of AceK shows the two-metal center and coordinating residues. Anomalous density is shown in orange and contoured at 4σ. Manganese ions are represented as purple spheres.

As expected, compared to the structure of AceK with Mg^2+^-ATP^23^, that of AceK-Mn^2+^ does not reveal large overall structural change (Fig. 1b). The two structures are superimposed with a root-mean-square deviation (RMSD) of 0.45 Å for the 554 Cα atoms. Previously, crystal structures of both AceK alone and AceK-IDH complex had been solved^23^. In these previous crystallization conditions, Mg^2+^ was added and only one metal ion was observed in the structures. In the current crystallization conditions, we added Mn^2+^ and found two Mn^2+^ ions in the active site of the structure. The only difference between previous and current conditions is the different metal ions added. For the two metal ions (designated M1 and M2) found in the catalytic center of AceK-Mn^2+^ the catalytic core displays an identical position of M1 compared to AceK-Mg^2+^. In the 2*F*_*o*_*-F*_*c*_-annealing OMIT map, well-defined electron density was visible for the dinuclear metal center (Fig. 2). Both sites were modeled as Mn^2+^ during crystallographic refinement, based on their octahedral coordination and the presence of MnCl_2_ only (no other metal) in the crystallization buffer. In order to confirm the presence of Mn^2+^ in the structure, spectrophotometric assay based on 4-(2-pyridylazo) resorcinol (PAR) was further performed and results showed the presence of Mn^2+^ and the estimated AceK/Mn^2+^ ratio was 1:2 (Fig. S1)^30,31^.

### A binuclear metal center at the catalytic site

**Figure 3:**
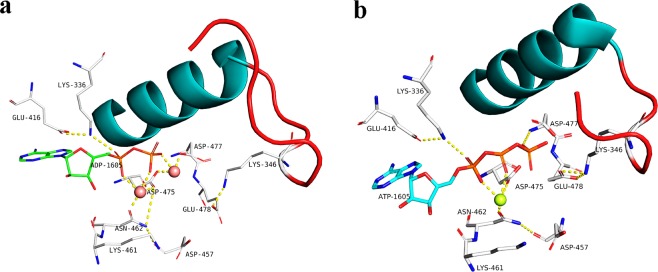
Catalytic center of AceK. (**a**) The manganese ion (salmon pink) binding sites are shown in the current AceK structure with ADP bound. The loop-β3αC is highlighted in the structure, in red. Interactions between Mn^2+^ ion and the residues in the binding pocket are shown as dashed lines. (**b**) The magnesium-binding site (green) in the previous AceK structure with ATP bound. Interactions between metal ions and the residues in the binding pocket are shown as dashed lines.

A binuclear Mn^2+^ ion site is formed in the catalytic site of the AceK-Mn^2+^ structure, similar to PPM or PPP phosphatases^4,32^. Two ions (M1, M2) separated by 3.9 Å are coordinated by the conserved aspartic acid residues Asp475, Asp477, and Asn462, ADP and water molecules (Fig. 3a). Each ion is hexa-coordinated by oxygen atoms from protein residues, ADP, and water molecules. The environments of the two metal ions in the AceK catalytic site differ (Fig. 3a); Mn^2+^ at M1 forms direct coordination with the carboxylate groups of two residues: Asp475, Asn462, which is the same with Mg^2+^ in the AceK-Mg^2+^ structure (Fig. 3b); while Mn^2+^ at M2 forms direct contact with carboxylate side chains of Asp477 and Asp475.

### Determination of the enzyme activity of AceK WT with Mg^2+^/Mn^2+^

**Figure 4:**
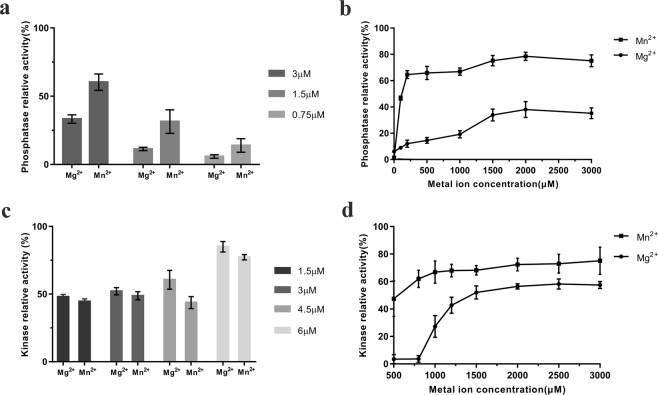
Enzymatic activity assay of AceK with Mn^2+^/Mg^2+^. (**a**) Relative phosphatase activity of AceK at three different protein concentrations (from left to right: 3.0 μM, 1.5 μM and 0.75 μM) with 2.0 mM MgCl_2_ or 2.0 mM MnCl_2_. Kinase inhibitor/phosphatase activator cocktail contains 5.0 mM AMP and 5.0 mM pyruvate. (**b**) Relative phosphatase activity of AceK with different concentrations of Mg^2+^or Mn^2+^; error bars indicate the standard deviations. (**c**) Relative kinase activity of AceK at four different protein concentrations (from left to right: 1.5 μM, 3.0 μM, 4.5 μM and 6.0 μM) with 2.0 mM MgCl_2_ or 2.0 mM MnCl_2_, and 2.0 mM ATP. (**d**) Relative kinase activity of AceK with different concentrations of Mg^2+^ or Mn^2+^; error bars indicate the standard deviations.

To assess the effects of different metal ions on the function of AceK, we measured the kinase and phosphatase activities of AceK in the presence of ADP, ATP, Mg^2+^ or Mn^2+^, using IDH and phospho-IDH as substrates. The relative phosphatase activities of AceK are shown in Fig. 4a. The phosphatase activity of AceK-Mn^2+^ was higher than AceK-Mg^2+^ at different enzyme concentrations. Without metal ions, the phosphatase activity of AceK was completely abolished (Fig. 4b). AceK was dependent on either Mg^2+^ or Mn^2+^ for catalytic activity because the phosphatase activities were higher with increasing concentrations of Mg^2+^ or Mn^2+^. When the concentration of Mg^2+^ was increased to 2.0 mM, the enzyme activity of AceK reached a maximum. Unlike AceK-Mg^2+^, AceK-Mn^2+^ displayed the highest phosphatase activity with the addition of 0.2 mM Mn^2+^. The maximal enzymatic activity stimulated by Mn^2+^ was 1- to 2-fold higher than that stimulated by Mg^2+^. In addition, Mn^2+^ stimulated protein phosphatase at a concentration 10-fold lower than that of Mg^2+^.

Mg^2+^ and Mn^2+^ had very similar effects on kinase activities (Fig. 4c). Although the enzyme concentration was increased from 1.5 μM to 6.0 μM the difference of kinase activity between AceK-Mg^2+^ and AceK-Mn^2+^ showed no great change. Without metal ions, the kinase activity of AceK was completely abolished (data not shown). As shown in Fig. 4d, the higher concentrations of Mg^2+^ significantly increased the kinase activities of AceK until enzyme activity was maximized with 2.0 mM Mg^2+^. AceK did not show any activity until the Mg^2+^ concentration reached 0.8 mM. Unlike AceK-Mg^2+^, AceK-Mn^2+^ exhibited appreciable kinase activity with the addition of 0.5 mM Mn^2+^ and reached the maximum at 2.0 mM Mn^2+^.

### AceK mutagenesis and effects on phosphatase activity

**Figure 5:**
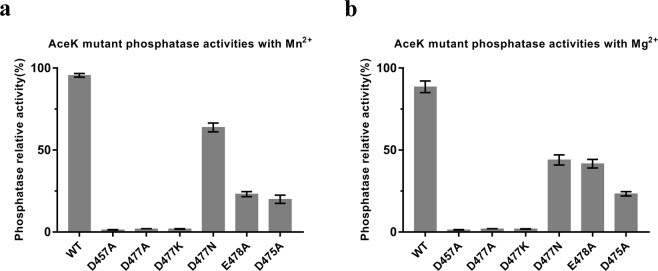
Phosphatase activity assay of AceK mutants with Mn^2+^/Mg^2+^. (**a**) Comparison of relative AceK phosphatase activity among AceK WT and the indicated mutants. (**b**) Comparison of relative AceK phosphatase activity among AceK WT and the indicated mutants, in the presence of the kinase inhibitor/phosphatase activator cocktail (5.0 mM AMP and 5.0 mM pyruvate). The assays were performed in the 20 mM Hepes buffer pH 7.0 containing 2.0 mM Mn^2+^/Mg^2+^ and 2.0 mM ADP at 37 °C. Three independent replicates were performed in every experiment; error bars indicate the standard deviations.

As shown in Fig. 4, the kinase activities of AceK in the presence of different metal ions did not appear to change much, whereas its phosphatase activities declined with Mg^2+^. As seen from the crystal structure, several key residues such as Asp457, Asp475, Asp477 and Glu478 are coupled with the Mn^2+^ ion and ADP to participate in AceK phosphatase catalysis (Fig. 3a). To investigate the effects of metal ions on the phosphatase activities, the AceK mutants of these catalytic residues were generated and the phosphatase activities in the presence of ADP were measured to compare with that of AceK wild-type (WT). Under standard assay conditions, the D457A, D477A and D477K mutants caused the complete loss of phosphatase activity, whereas E478A and D475A resulted in a decrease of phosphatase activity (Fig. 5). The loss of activity of mutants in M1 or M2 coordination indicates the pivotal role of the M1-M2 core in AceK phosphatase catalysis. Neither Asp457 nor Glu478 is involved in direct M1-M2 core coordination, and both residues are conserved among phosphatases. In the previous study, Asp457 was shown to serve as the proton donor in the phosphatase reaction in the presence of only one Mg^2+^ ion^28^. Meanwhile, the mutation of Asp457 caused the complete loss of phosphatase activity^28^. In comparison with D457A, the activity of E478A is slightly higher, which indicates that the role of Glu478 is important but not indispensable. In the structure of AceK-Mn^2+^, the M2 metal ion indirectly interacts with Glu478 through water-mediated hydrogen bonds. Therefore, the fact that the mutation of Glu478 with Mn^2+^ shows lower activity than that of Mg^2+^ demonstrates a more important role of Glu478 in the presence of Mn^2+^ which serves as the activating metal ion.

In the Mn^2+^-binding site of AceK, the two metal ions are both bridged by Asp475, whereas Asp477 is only coordinated to the M2 metal ion. However, in the Mg^2+^ binding site, only Asp475 directly coordinates M1. Therefore, we measured the phosphatase kinetics of the AceK WT, D475A, D477A and D477K mutants towards phospho-IDH in the presence of Mn^2+^ to further explore the catalytic role of the M1-M2 core. Our results showed that the *K*_m_ of D475A decreased by 5-fold, and the *K*_*cat*_/*K*_*m*_ decreased by approximately 24-fold in comparison with AceK WT (Table 1, Fig. S2). The D475A mutation in the presence of Mn^2+^ significantly reduced the phosphatase activities but had subtle effects on the *K*_*m*_ for phospho-IDH. We speculate that the mutation of D475A may perturb the metal ion center, changing the negative charge of the nucleophilic water and reducing its *K*_*cat*_. Conversely, the small effect of D475A on the *K*_*m*_ for phospho-IDH indicates that this residue does not affect the binding of the substrate or affect the overall protein structure.

**Table 1:** Kinetic constants of AceK and mutants with respect to phosphatase activities.

Protein	K_m_ (μM)	*K*_*cat*_ (s^−1^)	*K*_*cat*_/K_m_ (μM^−1^·s^−1^)	Ratio of *K*_*cat*_/K_m_ (WT/mutant)
WT	0.376 ± 0.054	122.6	326.1	1
D475A	1.946 ± 0.242	26.50	13.62	23.94
D477A	16.10 ± 2.650	21.88	1.359	240
D477K	12.17 ± 1.102	22.39	1.840	177.2

The assays were performed in the 20 mM Hepes buffer pH 7.0 containing 2.0 mM Mn^2+^ and 2.0 mM ADP at 37 °C. The *K*_*cat*_, *K*_*m*_ and *K*_*cat*_/*K*_*m*_ values were fitted to the equation 2 using GraphPadPrism5 software. The data were repeated at least three times.

Furthermore, a D477A mutation of AceK was generated, which eliminated the D477 side chain and abolished the indirect interaction between D477 and the M2 metal ion. To reverse the negative charge of the D477, we also constructed a D477K mutant to assess its effect on the enzymatic activity (Fig. S2). As shown in Table 1, the *K*_*m*_ of D477A and D477K decreased by 30- to 40-fold, and the *K*_*cat*_/*K*_*m*_ decreased by approximately 180- to 240-fold in comparison with AceK WT. In comparison with D475A, the activities of D477 mutants were drastically reduced. The activities of AceK were lost due to a highly reduced *K*_*m*_, which indicated that the substrate binding was affected. This may be the main reason that we could not obtain the crystal of the AceK D477A mutant in complex with phospho-IDH, despite persistent efforts.

### The interactions between Mn^2+^/Mg^2+^ and AceK in the presence of ADP or AMP

**Figure 6:**
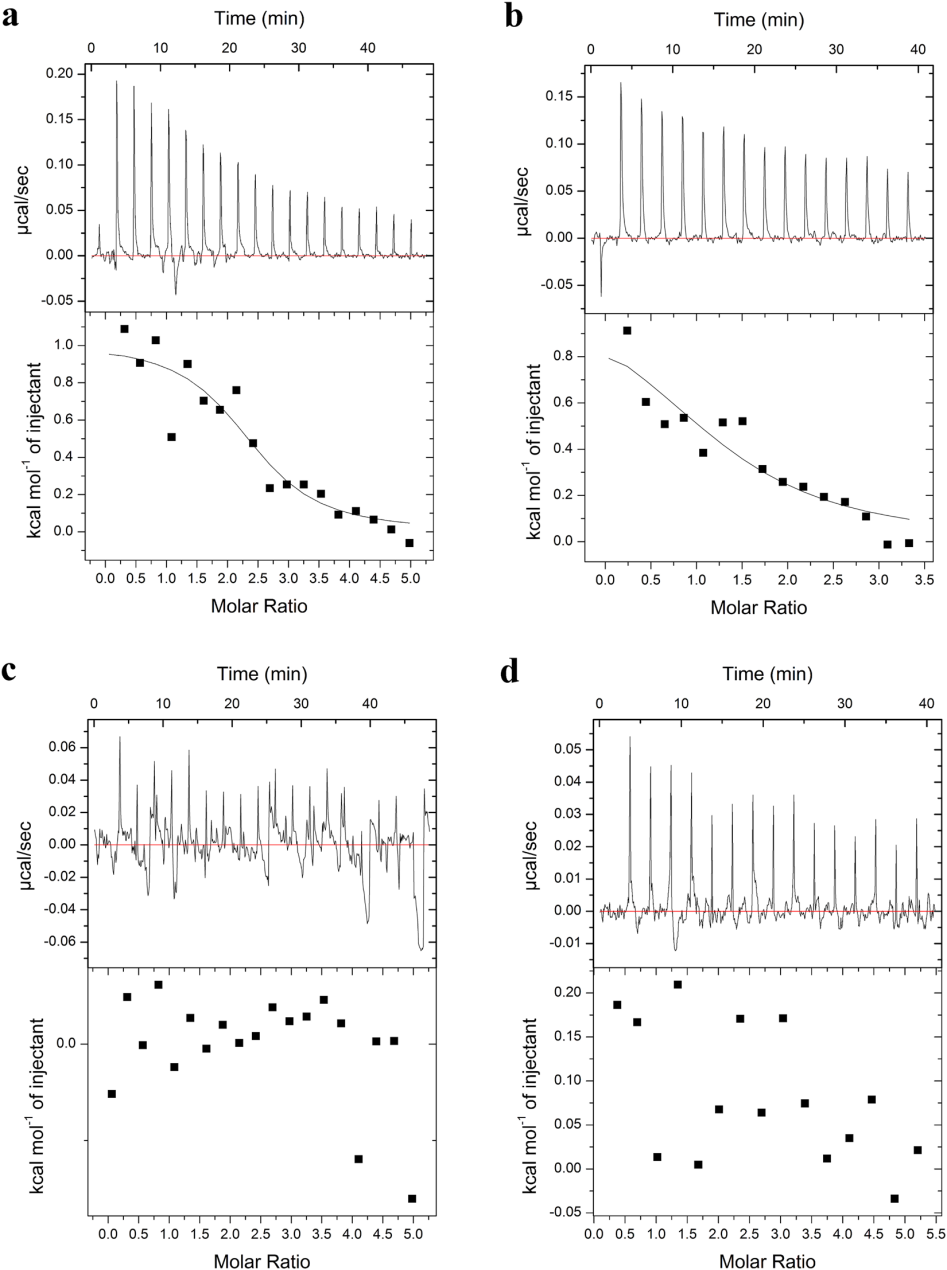
ITC titration curves of Mn^2+^and Mg^2+^ binding to AceK WT in the presence of ADP or AMP. For all titrations, upper panel: the raw data, and lower panel: corresponding binding isotherm fitted according to a single set of identical sites model, and the solid lines represents the best fit. (**a**) Binding of Mn^2+^ to AceK WT in the presence of ADP and AMP. (**b**) Binding of Mg^2+^ to AceK WT in the presence of ADP and AMP. (**c**) Binding of Mn^2+^ to AceK WT in the presence of AMP. (**d**) Binding of Mg^2+^ to AceK WT in the presence of AMP. The concentration of ADP and AMP was both 0.25 mM.

From the enzymatic experiments, we found that the mutation of residues coupled with metal ion resulted in the loss of phosphatase activity. Next, an ITC experiment was performed to determine the thermodynamic binding parameters of Mn^2+^ and Mg^2+^ for AceK WT and mutants under various conditions (Table 2). The ITC titration curves of AceK WT with Mn^2+^ and Mg^2+^ were obtained in the presence of ADP and AMP (Fig. 6a,b). Results showed that Mn^2+^ and Mg^2+^ bound to AceK WT with K_D_ values of 6.09 μM and 27.24 μM, respectively. The calculated numbers of binding sites were 2.37 for Mn^2+^ and 1.36 for Mg^2+^, which indicated that there is only one Mg^2+^ (presumably M1) binding to AceK WT, whereas two Mn^2+^ (presumably M1 and M2) binding to AceK WT. In addition, the affinity of Mn^2+^ for AceK WT was higher than that of Mg^2+^. The ITC results were consistent with our crystal structure. As seen from the crystal structures, ADP binds the metal ions. Therefore, we determined the binding affinities of Mn^2+^ and Mg^2+^ for AceK WT without ADP (Fig. 6c,d). Mn^2+^ and Mg^2+^ showed no binding affinities to AceK WT in the presence of only AMP. Thus, ADP plays an important role in the association with metal ions.

**Table 2:** Thermodynamic binding parameters for Mn^2+^and Mg^2+^ to AceK WT and the mutants from ITC experiments.

AceK-metal ion interactions	N	K_D_ (μM)	ΔH (kcal/mol)	ΔS (cal/mol/deg)
Mn^2+^-AceK WT with ADP and AMP	2.37	6.09	1.016	27.3
Mg^2+^-AceK WT with ADP and AMP	1.36	27.24	1.119	24.6
Mn^2+^-AceK WT with AMP	—*	—	—	—
Mg^2+^-AceK WT with AMP	—	—	—	—
Mn^2+^-AceK D477A with ADP and AMP	—	—	—	—
Mg^2+^-AceK D477A with ADP and AMP	—	—	—	—
Mn^2+^-AceK D477K with ADP and AMP	1.28	6.49	1.078	27.4
Mg^2+^-AceK D477K with ADP and AMP	—	—	—	—
Mn^2+^-AceK D475A with ADP and AMP	2.49	17.45	0.995	34.3
Mg^2+^-AceK D475A with ADP and AMP	—	—	—	—

*_ means not determined.

**Figure 7:**
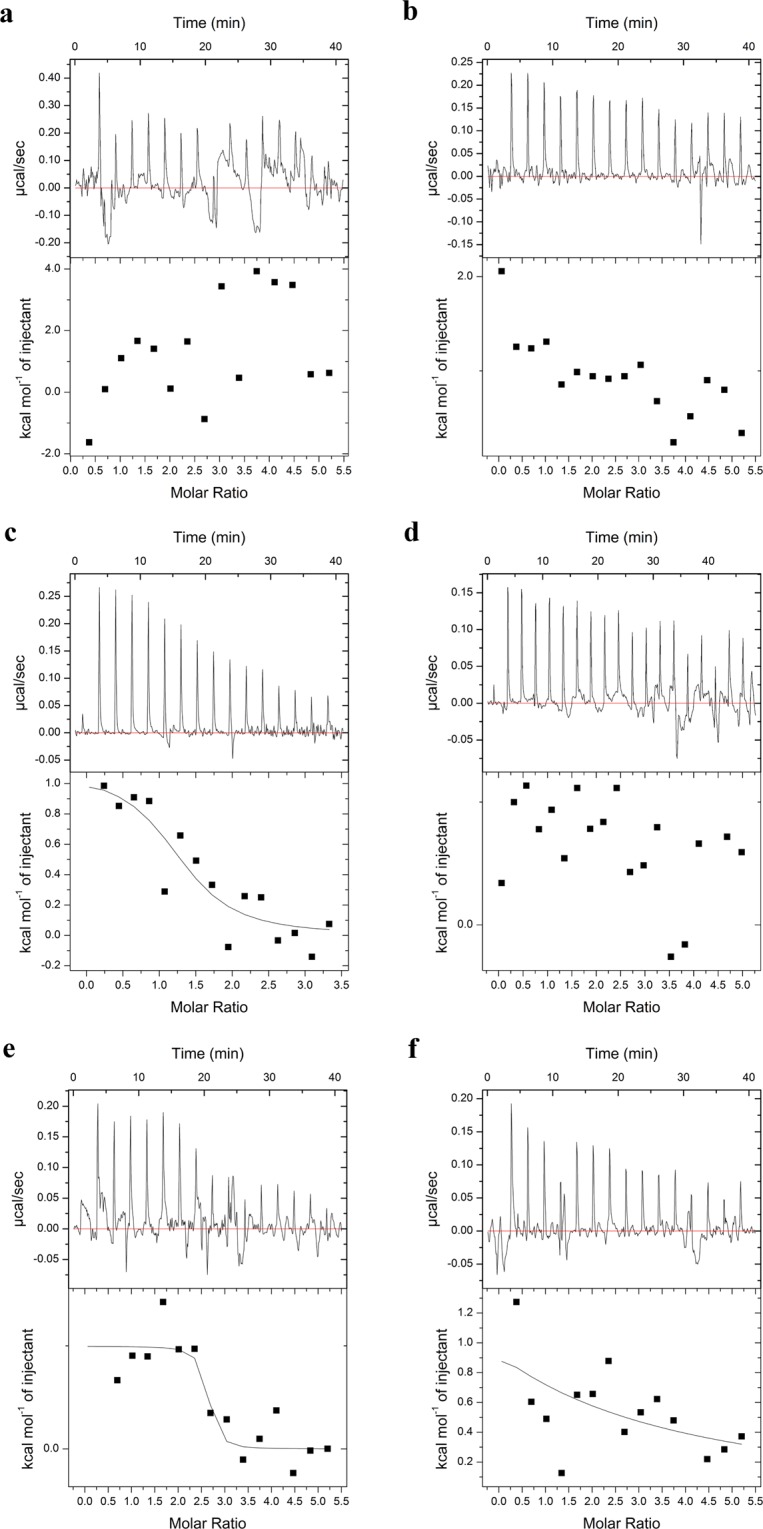
ITC titration curves of Mn^2+^ and Mg^2+^ binding to mutant AceK in the presence of ADP and AMP. (**a**) Binding of Mn^2+^ to AceK D477A (**b**) Binding of Mg^2+^ to AceK D477A (**c**) Binding of Mn^2+^ to AceK D477K (**d**) Binding of Mg^2+^ to AceK D477K (**e**) Binding of Mn^2+^ to AceK D475A (**f**) Binding of Mg^2+^ to AceK D475A. The concentration of ADP and AMP was both 0.25 mM.

Moreover, we assessed the interactions of AceK mutants with Mn^2+^and Mg^2+^ in the presence of ADP and AMP through ITC experiments (Table 1). As shown in Fig. 7a,b, AceK D477A could not bind to Mn^2+^and Mg^2+^. However, Mn^2+^ bound to AceK D477K with only one binding site, whose binding ratio and binding constant are 1.28 and 6.49 μM respectively, and Mg^2+^ showed no binding to AceK D477K (Fig. 7c,d). In the phosphatase analysis, the activities of D477 mutants were almost abolished in comparison with WT indicating that AceK enables phosphatase activity in the presence of two Mn^2+^ ions, but not one. Compared with WT, AceK D475A had two binding sites for Mn^2+^ with a lower binding constant of 17.45 μM. Moreover, the binding affinity was weak between AceK D475A and Mg^2+^ (Fig. 7e,f). Therefore, unlike D477 mutants, AceK D475A showed weak phosphatase activity in the presence of Mn^2+^.

### Cadmium inhibits dephosphorylation of phospho-IDH by AceK

**Figure 8:**
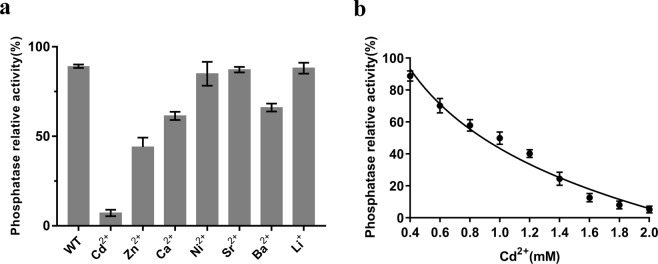
(**a**) The effects of metal ions on the phosphatase activities of AceK. All assays were performed in 20 mM Hepes, pH 7.0 buffer with 2.0 mM Mn^2+^. The concentration of metal ion was 2.0 mM. (**b**) Concentration dependence of the inhibition of AceK-catalyzed dephosphorylation of phospho-IDH by Cd^2+^. Three independent replicates were performed in every experiment; error bars indicate the standard deviations.

The PPM family phosphatases are a large group of Mg^2+^- or Mn^2+^-dependent Ser/Thr phosphatases. The role of Mn^2+^ in AceK is similar to the activating role of metal ions in PPM phosphatases. Some divalent metal ions, such as Cd^2+^, act as inhibitors in the hydrolysis of PPM phosphatases^33^. To determine whether these metal ions inhibit the phosphatase activity of AceK, we performed phosphatase assays of phospho-IDH in the presence of these divalent metal ions. Cd^2+^, Zn^2+^, Ca^2+^ and Ba^2+^ inhibited AceK, whereas other metal ions exhibited no influence (Fig. 8a). Similar to PPM phosphatases, among the metal ions screened, cadmium may be an inhibitor of the dephosphorylation of phospho-IDH by AceK. Next, we examined the effects of different concentrations of cadmium on the inhibition. As shown in Fig. 8b, when the concentration of Cd^2+^ was increased to 2.0 mM, the inhibition activity reached a maximum. AceK almost abolished phosphatase activity completely with the addition of 2 mM Cd^2+^.

## Discussion

Previous calculations revealed that AceK preferred a single metal ion for the kinase activity and that a second metal ion would unfavorably increase the reaction energy barrier^29^. In comparison, the presence of a single metal ion would lead to a higher energy barrier pathway for the phosphatase activity^28^. Previous theoretical studies and our current experiments indicated that M1 metal ion and M1-M2 core in the catalytic site had opposing effects on kinase and phosphatase activities. Our results of enzymatic assays demonstrate that AceK-Mn^2+^ showed higher phosphatase activity than AceK-Mg^2+^, whereas the kinase activity did not change much. Moreover, low concentrations of Mn^2+^ are more effective in activating phosphatase activity than low concentrations of Mg^2+^. Therefore, Mn^2+^ is likely to be the main metal ion controlling phosphatase activity.

To validate the effects of metal ions on phosphatase activity, we generated a number of mutants (D457A, D475A, D477A, D477K and E478A). The results of the decreased activities of mutants demonstrate the pivotal role of the M1-M2 core in AceK phosphatase catalysis. Combined with the kinetic parameters, we conclude that metal ion binding at the M2 site contributes to phospho-substrate binding. Asp477 has significant inhibitory influences on kinase activity as well as activating effects on phosphatase activity, which is mainly because that Asp477 is anchored to the M2 metal ion in the structure of AceK with Mn^2+^.

Notably, our ITC data indicate that there is only one Mg^2+^ binding to AceK WT, whereas two Mn^2+^ ions (M1 and M2) bind to AceK WT. This is in full agreement with the crystal structures. In addition, the binding of Mn^2+^ for AceK WT was stronger than that of Mg^2+^. Without ADP, Mn^2+^ and Mg^2+^ showed no binding to AceK WT, which demonstrates that ADP plays an important role in the association with metal ions, and AceK has a stronger interaction with two Mn^2+^ in the phosphatase model than one Mg^2+^. Combined with the ITC results of D477 mutants and the phosphatase catalysis, we show that AceK possesses phosphatase activity in the presence of two, but not one, Mn^2+^ ions.

Cadmium is a heavy metal with toxic effects in many organisms. The toxic mechanism of cadmium is complex, and one possibility is its affinity for metal proteins^34,35^. In the previous study, cadmium was discovered as a potent inhibitor for two PPM phosphatase members, PPM1A and PPM1G, but not PP1 or tyrosine phosphatases^33^. Furthermore, cadmium competitively binds the M1 binding site to inhibit PPM phosphatases^33^. Our results suggest that cadmium can inhibit the dephosphorylation of phospho-IDH by AceK. Therefore, we speculate that in the presence of two Mn^2+^, AceK dephosphorylates phospho-IDH in a way similar to PPM phosphatases.

In summary, to reveal the mechanism of AceK’s phosphatase activity, we determined the crystal structure of AceK in complex with Mn^2+^ and ADP. As shown in the crystal structures, there exist two Mn^2+^ ions (M1, M2) in the catalytic center of AceK, which is different from the previously studied structures containing only one Mg^2+^ ion. The binding of metal ions to AceK is directly responsible for enzyme activation. We suggest that Mn^2+^ is likely to be the main metal ion controlling phosphatase activity, and AceK possesses phosphatase activity only in the presence of two Mn^2+^ ions. In addition, ADP and D477 play the pivotal role in mediating metal binding of AceK.

Our findings suggest that metal binding in the AceK active site may serve a role to fine tune and balance kinase and phosphatase activities as one of the regulatory mechanisms. As AceK is a master regulator of cell metabolism, it is not surprising that its kinase/phosphatase bifunction is regulated through multiple mechanisms in response to nutrient availability. Our study provides more insights into further understanding the kinase and phosphatase function of AceK and its essential role in helping microorganisms cope with environmental stress.

## Material and Methods

### Construction of AceK mutants

All AceK mutants were constructed using PCR-mediated site directed mutagenesis method. The primers used for PCR amplification are shown in Table S2. The mutation was operated by PCR using KOD polymerase (TOYOBO). After purification by agarose gel electrophoresis and DNA purification kit (Tiangen), the PCR products were incubated with DpnI for 30 min. The digested products were next transformed into TOP10 and cultured on the agar LB medium plate overnight. A single clone was picked and the mutated sites were confirmed by DNA sequencing.

### Expression and purification of AceK mutants and IDH

Hexahistidine-tagged AceK mutants and IDH from *E*. *coli* were overexpressed in *E*. *coli* BL21 (DE3) cells. The frozen cells were resuspended in 50 ml lysis buffer containing 50 mM Tris-HCl, pH 7.5, 300 mM NaCl and 20 mM imidazole. Cells were lysed on ice by sonication. Cell debris was removed by centrifugation for 30 min at 18,000 g using a R20A2 rotor in a HITACHI high-speed centrifuge. The clarified lysate was applied onto Ni^2+^-NTA affinity resin (Qiagen) equilibrated with buffer A (50 mM Tris-HCl, pH 7.5, 300 mM NaCl and 20 mM imidazole) followed by a ten-column-volume wash in lysis buffer containing 40 mM imidazole. The protein was eluted in lysis buffer containing 300 mM imidazole.

The fractions containing protein were concentrated to 10 mg/ml using a Centricon-3 (Merck Millipore) and were further purified using an ÄKTA Purifier system (General Electric) with a size-exclusion HiLoad Superdex200 16/60 column in the buffer containing 20 mM HEPES, pH 7.0, 2.0 mM DTT, 100 mM NaCl and 10%(v/v) glycerol. Main protein fractions were pooled and concentrated to 8.0 mg/ml using a centrifugal filter unit (Amicon Ultra-15, 10,000, Merck Millipore).

### Crystallization of AceK with Mn

The purified AceK (66 kDa) was diluted to 5.0 mg/ml in a buffer consisting of 100 mM NaCl, 20 mM HEPES, pH 7.0, 2.0 mM DTT and 10%(v/v) glycerol. ADP was added to a final concentration of 1.0 mM and the hanging-drop vapor-diffusion method was used. Hanging drops contained 2 μL protein solution mixed with 2 μL well solution and 0.4 μL 2.0 mM MnCl_2_, which were equilibrated against 500 μL reservoir solution at room temperature. Tetragonal crystals appeared in 3 days and grew to full size within two weeks. The optimal crystallization conditions in the reservoir were 10%(v/v) glycerol, 2.0 mM DTT, 100 mM MES buffer, pH 6.0 with 4–10% PEG 8000 as the precipitating agent at room temperature. The reservoir solution itself was used as a cryoprotectant.

### Data collection, phasing and refinement

X-ray diffraction data were collected at 100 K with an oscillation angle of 1.0° over a total of 360°. The synchrotron data were indexed and integrated using HKL-3000^36^. The structure was determined by molecular replacement using *Phaser*^37^, using the structure of AceK extracted from AceK-IDH complex (PDB:3LCB) as a search model. The model was refined by manual building with *COOT*^38^ alternated with positional and B factor refinement with *Phenix*^39^. Structural figures were generated using *PyMOL* (http://www.pymol.org/). The atomic coordinates have been deposited in the Protein Data Bank (PDB:6K5L). The data collection and refinement statistics are summarized in Table S1.

### Activity assay

AceK kinase activity was assayed by coupling AceK activity to IDH activity, therefore AceK activity was measured indirectly by determination of IDH activity. AceK kinase activity was detected using a phosphorylation reaction system containing 20 mM Hepes-NaOH pH 7.0, 2.0 mM ATP, 2.0 mM MgCl_2_ or 2.0 mM MnCl_2_, 3.0 μM IDH and AceK. After incubating at 37 °C for 1 h, 20 μL of the mixture was added to 180 μL of reducing liquid (20 mM Hepes-NaOH pH 7.0, 2.0 mM threo-D,L isocitrate, 0.5 mM NADP^+^, 2.0 mM MnCl_2_), incubated at 37 °C for 15 min and terminated by 10 mM EDTA and 30 mM Na_2_CO_3_ solution. The activity of IDH was detected spectrophotometrically by monitoring the reduction of NADP^+^ at 340 nm using a PowerWave XS plate reader (Bio-Tek Instruments). Each component (buffer, AceK, each mutant and IDH alone) was also evaluated for their ability to reduce NADP^+^ as a control. All experiments were performed in triplicate. As the phosphorylation of IDH by AceK inhibits the activity of IDH, higher IDH activity corresponds to lower AceK kinase activity.

The phosphatase activity of AceK was measured as follows. We first performed the IDH phosphorylation reaction containing 2.0 mM MnCl_2_ by incubating the aforementioned phosphorylation reaction mixture for 12 h at 4 °C to ensure full phosphorylation of IDH. After 12 h, we removed Mn^2+^ from the reaction system by desalting column. Next, we added to the reaction solution 2.0 mM MgCl_2_ or 2.0 mM MnCl_2_, 2.0 mM ADP, 5.0 mM AMP and 5.0 mM pyruvate that inhibits kinase activity and activates phosphatase activity. After incubation for 30 min at 37 °C, the activity of IDH was reactivated and dephosphorylated. Next, 20 μL of this mixture was added to 180 μL reducing solution and incubated at 37 °C for 15 min before termination, followed by the IDH activity assay. Corresponding samples without AMP and pyruvic acid were also used to show that AMP and pyruvic acid has no effect on IDH. The other reference samples were used in the same experiment conditions. All experiments were performed independently in triplicate. We performed AceK kinase assays by similarly monitoring the reduction of NADP^+^ to NADPH at 340 nm. As dephosphorylation of IDH by AceK removes inhibition of the activity of IDH, lower AceK phosphatase activity results in lower IDH activity.

### Kinetic analysis

To determine the kinetic parameters of *K*_*cat*_ and *K*_*ca*t_/*K*_*m*_, the reactions were performed in a reaction buffer (20 mM Hepes pH 7.0 with 5.0 mM AMP and 5.0 mM pyruvate) containing 2.0 mM Mn^2+^ and ADP at 37 °C. Then the reactions were terminated by 10 mM EDTA and 30 mM Na_2_CO_3_ solution and assessed by measuring the reduction of NADP^+^ to NADPH at 340 nm. One unit of enzyme is defined as the amount of the enzyme required for the hydrolysis of 1 μM substrate per minute at 37 °C.

### Spectrophotometric analysis of manganese content

A method for the spectrophotometric determination of manganese content was adapted from a previously published protocol^30,31^. AceK protein sample was prepared at a concentration of 150–200 μM. Each protein sample, in addition to 1 mM manganese chloride was incubated for 1 h at 4 °C. Then we removed extra Mn^2+^ from the system by using desalting column to obtain the sample AceK-Mn^2+^ sample. 10 μl of the AceK-Mn^2+^ sample was added to 80 μl of 100 μM 4-(2-pyridylazo)-resorcinol (PAR) in 20 mM phosphate, pH 11.2, incubated for 1 h at room temperature, and UV absorbance spectra were recorded between 600 and 300 nm (Varian Cary 50 spectrophotometer). Manganese concentrations were estimated by comparison of A492 nm with lines of best fit obtained from analysis of 100–900 μM manganese chloride solutions. Protein concentrations were determined by UV spectrophotometry, with extinction coefficients and molecular weights calculated by ProtParam (http://web.expasy.org/protparam/).

### Isothermal titration calorimetry (ITC)

All ITC experiments were performed using a VP-ITC instrument (GE MicroCal) at 25 °C. Proteins were purified using buffer containing 20 mM Hepes pH 7.0 and the metal ions were dissolved in the same buffer. 40 µM AceK proteins (WT, D477A, D477K and D475A) was titrated using 1.0 mM Mn^2+^ or Mg^2+^ in the presence of 0.25 mM ADP or AMP. Experiments were done in triplicate. The baseline and binding parameters (binding site number (N), binding constant (Ka), and change in enthalpy of binding (ΔH)) were generated using the MicroCal Origin package.

### Cadmium inhibition assay

The dephosphorylation of phospho-IDH was performed in a reaction buffer containing 20 mM Hepes pH 7.0, 5.0 mM AMP, 5.0 mM pyruvate and 2.0 mM Mn^2+^ and ADP at 37 °C, with or without 2.0 mM Cd^2+^. Then the reactions were terminated by 10 mM EDTA and 30 mM Na_2_CO_3_ solution and assessed by measuring the reduction of NADP^+^ to NADPH at 340 nm.

### Accession number

Data deposition: The atomic coordinates have been deposited in the Protein Data Bank, www.rcsb.org (PDB:6K5L).

## Supplementary information

Characterization of metal binding of bifunctional kinase/phosphatase AceK and implication in activity modulation

## Data Availability

The datasets generated during and/or analysed during the current study are available from the corresponding author on reasonable request.
