# Author Correction: Characterization of metal binding of bifunctional kinase/phosphatase AceK and implication in activity modulation

**DOI:** 10.1038/s41598-020-60917-3

**Published:** 2020-03-06

**Authors:** Xiaoying Zhang, Qingya Shen, Zhen Lei, Qianyi Wang, Jimin Zheng, Zongchao Jia

**Affiliations:** 1 College of Chemistry, Beijing Normal University, Beijing, 100875 China; 2 Department of Biomedical and Molecular Sciences, Queen’s University, Kingston, Ontario K7L3N6 Canada

Correction to: *Scientific Reports* 10.1038/s41598-019-45704-z, published online 24 June 2019

This Article contains typographical errors.

In the Methods section the subheading,

‘Crystallization of AceK with Mn’

should read:

‘Crystallization of AceK with Mn^2+^’

In the Acknowledgements section,

“We would like to thank the staff at the BL17U of Shanghai Synchrotron Radiation Facility (SSRF) for X-ray data collection. We are grateful to Dr. Guohua Jiang from College of life science, Beijing Normal University for his help with ITC experiments. This work was supported by grants from the National Natural Science Foundation of China (No.21133003 and No.21773014) and the Canadian Institutes of Health Research.”

should read:

“We would like to thank the staff at the BL17U of Shanghai Synchrotron Radiation Facility (SSRF) for X-ray data collection. We are grateful to Dr. Guohua Jiang from College of Life Sciences, Beijing Normal University for his help with ITC experiments. This work was supported by grants from the National Natural Science Foundation of China (No.21133003 and No.21773014) and the Natural Sciences and Engineering Research Council of Canada (RGPIN-2018-04427).”

