# Supplementary material for: Characterization of metal binding of bifunctional kinase/phosphatase AceK and implication in activity modulation
Source: Sci Rep. 2019 Jun 24;9:9198. doi: 10.1038/s41598-019-45704-z (PMC6591243; doi:10.1038/s41598-019-45704-z)
Supplement: Supplementary file 1 — Characterization of metal binding of bifunctional kinase/phosphatase AceK and implication in activity modulation [file 41598_2019_45704_MOESM1_ESM.pdf]

Supplementary Information

Characterization of metal binding of bifunctional kinase/phosphatase AceK and implication in activity modulation

Xiaoying Zhang[1], Qingya Shen[1], Zhen Lei[1], Qianyi Wang[1], Jimin Zheng[1,*] and Zongchao Jia[2,*]

[1] College of Chemistry, Beijing Normal University, Beijing, 100875, China
[2] Department of Biomedical and Molecular Sciences, Queen's University, Kingston, Ontario, K7L3N6, Canada

*** Correspondence:**
Jimin Zheng; Zongchao Jia
Email: jimin_z@bnu.edu.cn (JZ); jia@queensu.ca (ZJ)

Supplementary information

A supplementary PDF file contains results of spectrophotometric analysis of manganese content (Figure S1), kinetic analysis of wild type and mutant AceK (Figure S2), statistics of X-ray diffraction data and structure refinement (Table S1) and primers of mutants used in this study (Table S2).

Supplementary Figures

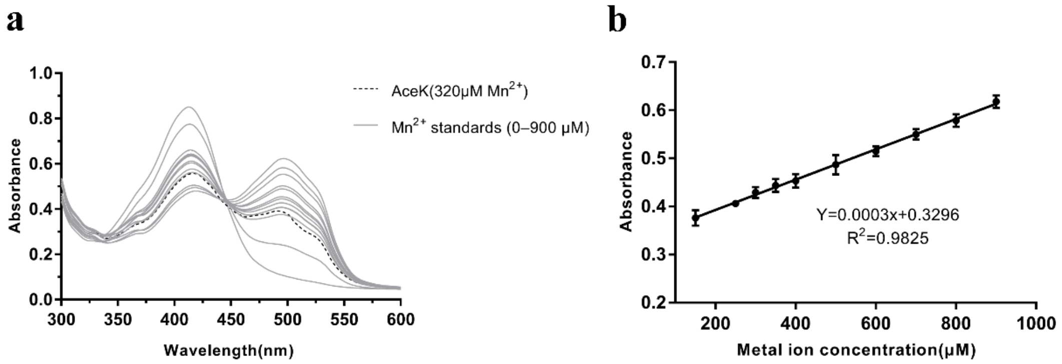

Figure S1. Spectrophotometric determination of manganese content for AceK. (a) Manganese content for AceK (dashed line) was determinated using metal chelator 4-(2-pyridylazo) resorcinol (PAR), with manganese standards shown in grey. (b) The standard curve showed the absorbance of different concentrations of manganese.

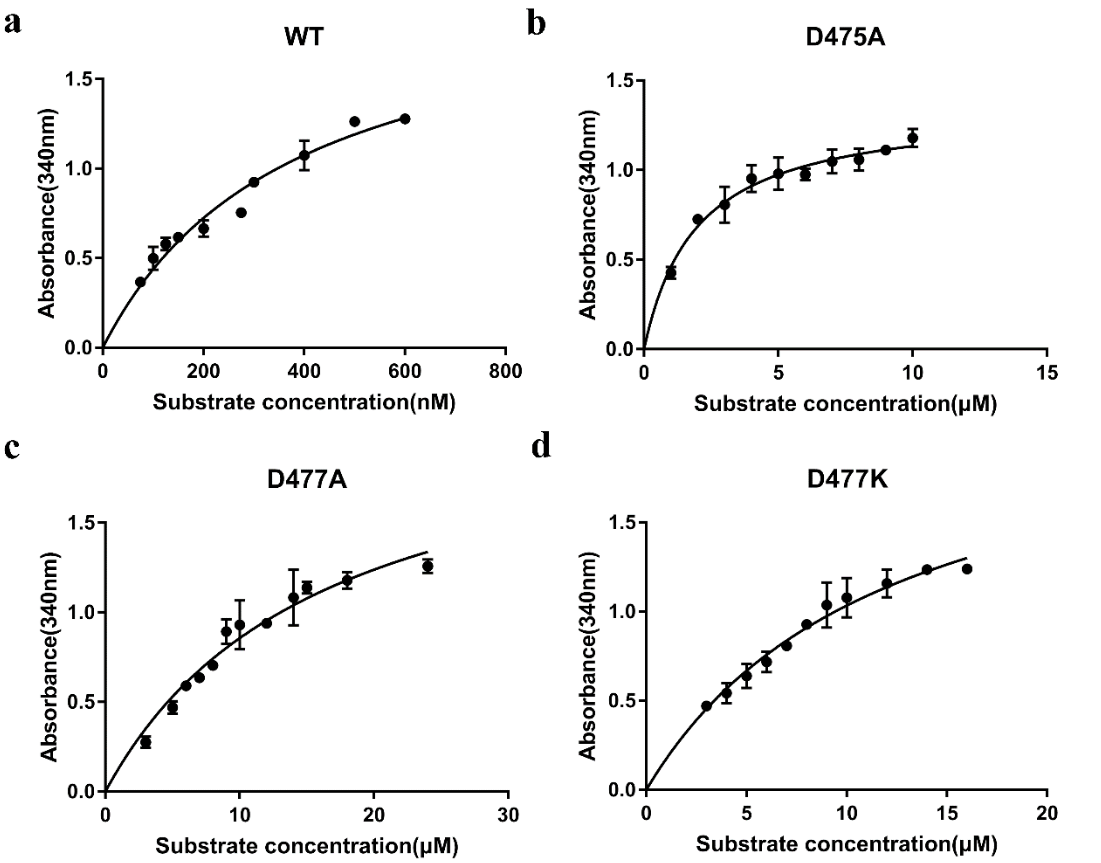

Figure S2. Kinetic analysis of wild type and mutant AceK (D475A, D477A and D477K) using varying concentrations of phospho-IDH, 2.0 mM Mn2+ and 2.0 mM ADP. Three independent replicates were performed in every experiment; error bars indicate the standard deviations.

Supplementary Table

Table S1. Statistics of X-ray diffraction data.

Protein form	AceK
Wavelength (Å)	0.979
Space group	$P4_12_12$
Unit-cell parameters (Å)	a = b = 124.51, c = 266.85
Resolution (Å)	30 -2.55
Total number of reflections	560797 (55768)
Number of unique reflections	69363 (4784)
Completeness (%)	97 (100)
$<I/\sigma>$	23.00 (2.12)
$^{‡}R_{work}$ /$^{§}R_{free}$ (%)	18.95(21.94)
Molecules per AU	2
Solvent content	0.40
R.m.s.d bond length (Å)	0.004
R.m.s.d bond angle (°)	0.77
Ramachandran(%)	
most favoured	96
allowed	3.9
disallowed	0.09
Ligand and metal	ADP, AMP, Mn^{2+}
PDB ID	6K5L

$^{‡}R_{work} = \Sigma||F_o| - |F_c||/\Sigma|F_o|$.

$^{§}R_{free}$ calculated with 10% of all reflections excluded from refinement.

Values in parentheses are for the highest resolution shell.

Table S2. The primers of AceK mutants for PCR amplification

Mutant	Primer	Sequence 5’-3’
D457A	Sense	TAACATTTTCCCGGGCGCCATGCTGTTTAAAAAC
	Antisense	GTTTTTAAACAGCATGGCGCCCGGGAAAATGTTA
D475A	Sense	GCGTGTGGTTTTTTATGCCTACGATGAAATTTGC
	Antisense	TGTAGCAAATTTCATCGTAGGCATAAAAAACCAC
D477A	Sense	TGTGGTTTTTTATGATTACGCCGAAATTTGCTACATG
	Antisense	CTTCCGTCATGTAGCAAATTTCGGCGTAATCATAAAA
D477K	Sense	TGTGGTTTTTTATGATTACAAAGAAATTTGCTACATG
	Antisense	CTTCCGTCATGTAGCAAATTTCTTTGTAATCATAAAA
D477N	Sense	TGTGGTTTTTTATGATTACAACGAAATTTGCTACATG
	Antisense	CTTCCGTCATGTAGCAAATTTCTTGGTAATCATAAAA
E478A	Sense	TGGTTTTTTATGATTACGATGCAATTTGCTACATGACGG
	Antisense	CCGTCATGTAGCAAATTGCATCGTAATCATAAAAAACCACA
